# Development of a parent‐reported questionnaire evaluating upper limb activity limitation in children with cerebral palsy

**DOI:** 10.1002/pri.1684

**Published:** 2017-01-23

**Authors:** N. Preston, M. Horton, M. Levesley, M. Mon‐Williams, R.J. O'Connor

**Affiliations:** ^1^ Academic Department of Rehabilitation Medicine, Faculty of Medicine and Health University of Leeds Leeds United Kingdom of Great Britain and Northern Ireland; ^2^ School of Mechanical Engineering University of Leeds Leeds United Kingdom of Great Britain and Northern Ireland; ^3^ School of Psychology, Faculty of Medicine and Health University of Leeds Leeds United Kingdom of Great Britain and Northern Ireland; ^4^ National Demonstration Centre in Rehabilitation Leeds Teaching Hospitals NHS Trust Leeds United Kingdom of Great Britain and Northern Ireland; ^5^ Academic Department of Rehabilitation Medicine, Leeds Institute of Rheumatic and Musculoskeletal Medicine, School of Medicine, Faculty of Medicine and Health University of Leeds Leeds United Kingdom of Great Britain and Northern Ireland

**Keywords:** cerebral palsy, measurement, paediatrics, Rasch, upper limb function

## Abstract

**Background and purpose:**

Upper limb activity measures for children with cerebral palsy have a number of limitations, for example, lack of validity and poor responsiveness. To overcome these limitations, we developed the Children's Arm Rehabilitation Measure (ChARM), a parent‐reported questionnaire validated for children with cerebral palsy aged 5–16 years.

This paper describes both the development of the ChARM items and response categories and its psychometric testing and further refinement using the Rasch measurement model.

**Methods:**

To generate valid items for the ChARM, we collected goals of therapy specifically developed by therapists, children with cerebral palsy, and their parents for improving activity limitation of the upper limb. The activities, which were the focus of these goals, formed the basis for the items. Therapists typically break an activity into natural stages for the purpose of improving activity performance, and these natural orders of achievement formed each item's response options. Items underwent face validity testing with health care professionals, parents of children with cerebral palsy, academics, and lay persons.

A Rasch analysis was performed on ChARM questionnaires completed by the parents of 170 children with cerebral palsy from 12 hospital paediatric services. The ChARM was amended, and the procedure repeated on 148 ChARMs (from children's mean age: 10 years and 1 month; range: 4 years and 8 months to 16 years and 11 months; 85 males; Manual Ability Classification System Levels I = 9, II = 26, III = 48, IV = 45, and V = 18).

**Results:**

The final 19‐item unidimensional questionnaire displayed fit to the Rasch model (chi‐square *p* = .18), excellent reliability (person separation index = 0.95, α = 0.95), and no floor or ceiling effects. Items showed no response bias for gender, distribution of impairment, age, or learning disability.

**Discussion:**

The ChARM is a psychometrically sound measure of upper limb activity validated for children with cerebral palsy aged 5–16 years. The ChARM is freely available for use to clinicians and nonprofit organisations.

## 1 INTRODUCTION

Cerebral palsy is a clinical diagnosis characterised by disorders of movement, posture, and motor function (Odding, Stam, & Hendrik, 2006). Up to 80% of children with cerebral palsy experience upper limb motor impairment (Cans et al., 2007), causing activity limitation (e.g., difficulty with washing, eating, or preparing meals [World Health Organisation, 2016]).

Traditional interventions improve the independence of children with cerebral palsy by addressing activity limitation, that is, improving active function by the independent movement of the child to achieve an activity (Ashford & Turner‐Stokes, 2013). In recent years, research and reviews investigating these interventions suggest that there is a lack of valid and responsive measures for evaluating changes in upper limb activity limitation (Hoare et al., 2010; Meyer‐Heim & van Hedel, 2013; Palsbo & Hood‐Szivek, 2012; Qiu et al., 2009; Sakzewski, Ziviani, & Boyd, 2009; Sandlund, Mcdonough, & Hager‐Ross, 2009). This is supported by systematic reviews into measures of activity limitation for children with cerebral palsy (Gilmore, Sakzewski, & Boyd, 2010; Greaves, Imms, Dodd, & Krumlinde‐Sundholm, 2010; Klingels et al., 2010). These reviews suggest that the ABILHAND‐Kids is the most psychometrically robust measure available for this purpose.

The ABILHAND‐Kids has been developed using Rasch analysis, which allows the transformation of ordinal outcome scores into linear (interval‐level) scores if the data from their items fit the Rasch mathematical model (Bond & Fox, 2015, p. 29). This approach satisfies the compelling argument that ordinal outcome scores should not be used in clinical trials (Hobart, Cano, Zajicek, & Thompson, 2007; Merbitz, Morris, & Grip, 1989). However, there is no evidence that the ABILHAND‐Kids measure is responsive (Gilmore et al., 2010; Greaves et al., 2010). Other studies using the ABILHAND‐Kids also suggest a lack of responsiveness (Preston et al., 2016; Preston et al., 2015). The adult version of the ABILHAND‐Kids (the ABILHAND) also has limited responsiveness when compared with other measures (Bovolenta, Clerici, Agosti, & Franceschini, M, 2009). Additionally, the ABILHAND‐Kids was validated on a sample of French‐speaking children with cerebral palsy that included only four children with severe activity limitation, on which a floor effect was reported, and 46% of the remaining sample were classed as having minimal to no activity limitation (Arnould, Penta, Renders, & Thonnard, 2004). It is increasingly important that the validity and scale range of measures of activity limitation include children with more severe disability, because new approaches such as robotic and computer‐assisted rehabilitation technology are potentially more inclusive for children whose degree of disability prevents their participation in other rehabilitation practices such as constraint‐induced movement therapy (Fasoli et al., 2010, Meyer‐Heim & van Hedel, 2013). Since the reviews of Gilmore et al. (2010), Greaves et al. (2010), and Klingels et al. (2010), two other measures with good potential (the paediatric motor activity log [revised; Wallen, Bundy, Pont, & Ziviani, 2009] and the Children's Hand‐use Experience Questionnaire [Skold, Krumlinde‐Sundholm, Hermansson, & Eliasson, 2009]) have been developed using Rasch analysis, but they still require further psychometric testing (Skold, Hermansson, Krumlinde‐Sundholm, & Eliasson, 2011; Wallen & Ziviani, 2013), and some items appear unsuitable for all children, for example, fastening a necklace (Skold et al., 2011). They are not validated for use outside of their respective countries (Australia and Sweden).

Irrespective of whether potential benefits to upper limb activity limitation are being evaluated after experimental or clinical interventions, our experience and investigations into available measures for evaluating upper limb activity limitation suggested that a new measure validated for children with cerebral palsy in the UK was necessary. Because current clinical and experimental approaches have the potential to benefit both unilateral and bilateral activity, we saw no advantages in a measure that evaluates only unilateral or only bilateral upper limb activity limitation. We defined activities for the new measure as those upper limb activities listed within the International Classification of Function, Health, and Disability for Children and Youth developed by the World Health Organisation (http://apps.who.int/classifications/icfbrowser/) and set out to construct a measure that encompasses the most common activities of daily living at which children with cerebral palsy experience limitation. By developing the new measure using the Rasch model, we intended that the final measure would permit transformation of the raw scores to interval‐level measurement.

The Rasch model is a probabilistic mathematical model of measurement based upon, but less rigid than, the (deterministic) Guttman pattern (Bond & Fox, 2015 p. 177; Tennant & Conaghan, 2007). The underlying principle for constructing measures based on the Rasch model is that the probability of a person endorsing, or “passing,” an item is influenced only by the difficulty of the item and the ability of the person (Tennant & Conaghan, 2007). Endorsing an item illustrates a specific “quantity” of the trait being measured, and it is probable that all easier items will be also be endorsed by that person. This technique allows the person being measured to be numerically quantified on a logistic scale if the items themselves are on a linear scale and if they are unidimensional (they all relate strongly to the trait being measured and not a different underlying trait). The linear (interval) scales on which items and persons are numerically located are calibrated in log‐odds units called logits. These units represent the natural logarithm of the odds of success, that is, endorsing (or passing) an item (Bond & Fox, 2015 p. 29).

Responses to items showing a good fit to the Rasch model are determined to have met the fundamental principles of measurement for achieving linear (interval‐level) outcome scores (Newby, Conner, Grant, & Bunderson, 2009). Bond & Fox (2015, Chapter 3) give a helpful description of these principles, and a commentary of what should be expected from a Rasch analysis is provided by Tennant and Conaghan (2007).

This study therefore aimed to develop and establish a psychometrically sound, parent‐completed questionnaire for measuring activity limitation of children with cerebral palsy aged 5–16 years that could produce interval‐level measurement and that had no floor effects even in children with the most severe upper limb activity limitation.

## 2 METHODS

### 2.1 Ethical approval and funding

Ethical approval was received from the East Yorkshire and North Lincolnshire Research Ethics Committee (REC Reference 10/H1304/46). Consent to participate was implied by parents returning a completed questionnaire. The trial was registered on the National Institute for Health Research portfolio (ID 9600). The study was an educational project funded by the National Institute for Health Research under their Doctoral Fellowship programme.

### 2.2 Item and response category development

To develop appropriate items for the new measure, we used an approach, which aimed to focus the items squarely within the dimension of activity limitation, and specifically those activities at which children with cerebral palsy most commonly experience limitations. Our hypothesis for this approach was that treatment goals targeting upper limb activity limitation, formulated after functional assessment of children with cerebral palsy aged 5–16 years by clinical and research doctors and therapists, would provide an appropriate basis for items, which relate directly to upper limb activity. We approached 14 therapy teams across England to collect appropriate goals of rehabilitation therapy. We combined these with goals taken from our own research work (Preston et al., 2016; Weightman et al., 2011; Preston, Clarke, & Bhakta, 2011). For the purposes of the Children's Arm Rehabilitation Measure (ChARM's) item set, the goals were rewritten to form item stems. A major advantage of this approach is that the completed measure will have great clinical relevance because it is based on the most common functional difficulties experienced by the population for whom the measure is validated.

Response options for items also need to be properly developed. Item responses can be varied in type or number (Streiner & Norman, 2003, pp. 33–35), or they can be consistent for each item, for example, rating capability as “Easy, Difficult, or Impossible” for each item, as in the ABILHAND‐Kids. Too many response options can introduce error (Bond, 2003). Conversely, too few response options may result in poor responsiveness (Bovolenta et al., 2009), possibly as a consequence of increased floor and ceiling effects caused by the width of the categories (Merbitz et al., 1989).

Bond and Fox (2015, p. 160) suggest that the optimum number of response options is entirely dependent on the characteristic being measured and should be assessed empirically for each scale. We therefore elected to develop item responses from the natural stages into which each item's activity can be broken as is typically done in rehabilitation by therapists working on reducing activity limitation (Bobath, 1990). For example, the item responses for the item “donning a vest” included the following natural stages:

- Yes, my child can put on a vest.
- My child can put on a vest if it is laid out first.
- My child can put on a vest once it has been pulled over their head or one arm.
- My child can complete putting on a vest once it has been put on over the head and arms.
- No, my child needs help to completely put on a vest.

Individual items therefore had a differing number of response options. The resulting item set was reviewed by between two and five therapists spread across the 12 rehabilitation teams that agreed to support the development of the ChARM. The item set was then formulated into the ChARM questionnaire.

The ChARM underwent face validity testing by a process in which the ChARM was reviewed by five groups of five or six people, one group after another. Each group included paediatric therapists, parents of children with cerebral palsy, professors and researchers who specialise in psychometrics and in the development of new measures, and lay persons. After each group's review, the reviewers' comments were addressed before the ChARM was reviewed by the next group of reviewers. The process was repeated four times in total. Paediatric therapists were not from the teams that had been involved in the generation of goals or the review of the items.

The aim of the next stage was to obtain a dataset of ChARM responses in order to perform psychometric testing. This required parents of children with cerebral palsy aged 5–16 years across the range of manual disability treated by paediatric therapists (Manual Ability Classification System [MACS; Eliasson et al., 2006] Levels II–V) to complete the ChARM and return it to us. Therefore, the paediatric therapy teams posted to the parents of each child on their caseload that met these criteria a ChARM, an information sheet and a prepaid, addressed envelope for parents to return the ChARM directly to the research team. A web‐based version of the questionnaire was available for parents who preferred to submit responses online. Both versions included a section for parents to give details of their child for the purposes of investigating response bias, for example, gender, age, and manual ability. We also included a text box for parents to leave comments.

Following an initial Rasch analysis on this first draft of the ChARM, we modified the ChARM on the basis of the Rasch findings and posted this ChARM version 2 back to the parents that had returned the first draft in order to perform a second Rasch analysis. To overcome the possibility that we would not receive a response from every family in the original cohort, therapy teams from two additional regional paediatric services posted out the questionnaire to the parents of children with cerebral palsy aged 5–16 years. We also used social media (e.g., Facebook and the message boards of Hemiplegia and Scope) to attempt to increase the sample of children with cerebral palsy for whom the ChARM would be completed.

### 2.3 Rasch analysis

The Rasch analyses in this study were performed using RUMM2030 Version 5.4 for Windows, Copyright 1997–2012 RUMM Laboratory Pty Ltd. Masters' Partial Credit Model (unrestricted; polytomous or extended response category test format) was used because item responses varied in type and number between items (Masters, 1982). The analyses generate summary statistics illustrating mean person and item locations and the overall fit to the Rasch model based on a chi‐squared test of fit. Additionally, two measures of internal consistency are available: the person separation index (PSI) and Cronbach's α. In order to power an adequate Rasch analysis, we required a minimum of 100 completed ChARMs to achieve 95% confidence of item calibration to within 0.5 logits (Linacre, 1994). We did not collect data on how many ChARMs were posted by therapy teams.

Individual item analysis includes an assessment of individual item fit (using chi‐square and standardised fit‐residual statistics), response category threshold ordering, response dependency, and item response bias (differential item functioning). Additionally, unidimensionality is investigated by identifying the two most divergent subsets of items within the first factor of a principal component analysis of the residuals, as described in Tennant and Conaghan (2007). Separate person estimates are generated for each of these divergent item subsets, and differences in the individual person estimates are evaluated using a series of *t* tests. The percentage of significant tests should not exceed 5%, and the lower bound confidence interval for a binomial test of proportions should overlap (i.e., be lower than) the 5% limit to indicate unidimensionality.

Where disordered thresholds are present, amendments will be made by combining two or more adjacent response categories. Where evidence of response dependency or multidimensionality is present, items will be removed.

Once fit to the model is achieved, each deleted item will be individually reintroduced to the final item set to reevaluate the initial source of misfit.

To evaluate external construct validity of the ChARM, we hypothesised that there would be significant differences between mean logit scores of all children grouped by MACS (manual ability) level. To determine this, we planned to perform an analysis of variance on mean logit scores calculated for all children within each MACS level.

## 3 RESULTS AND FINDINGS

### 3.1 Initial Rasch analysis

The initial Rasch analysis was conducted on a dataset from 170 ChARMs, each with 40 items, completed by the parents of children with cerebral palsy who were approached anonymously through the 12 regional therapy teams. This revealed a number of psychometric problems, for example, misfitting items and lack of unidimensionality. We addressed these problems through a process that is described in more detail below. The initial Rasch analysis informed development of ChARM draft 2, which showed good fit to the Rasch model but also a large floor effect (greater than 20% of scores outside the range of the scale (Holmes & Shea, 1997)). One parent of a MACS Level V child listed 10 items in the comments section, which she suggested were missing but desirable for her child. Six of these items were relevant for both age range and gender and were added to ChARM draft 2 in an attempt to address the floor effect. This resulted in a questionnaire of 25 items, which was sent out to parents to obtain a new dataset on which to perform a second Rasch analysis and develop a final version of the ChARM. We received a completed 25‐item ChARM draft 2 from 148 parents of children whose demographics and clinical details are given in Table 1. None was a result of the use of social media. All data were included in the psychometric testing.

**Table 1:** Demographics and clinical details of sample used to validate the ChARM

Demographics (*n* = 148)		
Age in years and months	Mean (SD)	10 years and 1 month (3 years and 3 months)
	Median	11 years and 9 months
	Min	4 years and 8 months
	Max	16 years and 11 months
Gender	Male	85 (57%)
	Female	57 (39%)
	Missing data	(6) (4%)
MACS Levels	Level I	9 (6%)
	Level II	26 (18%)
	Level III	48 (32%)
	Level IV	45 (30%)
	Level V	18 (12%)
	Missing data	2 (2%)
Distribution	Bilateral	77 (52%)
	Unilateral	59 (40%)
	Lower limb only^a^	12 (8%)
Learning impairment	Present	85 (57%)
	Not present	61 (41%)
	Missing data	2 (2%)
Visual impairment	Present	62 (57%)
	Not present	84 (39%)
	Missing data	2 (2%)
Hearing impairment	Present	18 (12%)
	Not present	128 (86%)
	Missing data	2 (2%)
Speech impairment	Present	72 (48%)
	Not present	74 (50%)
	Missing data	2 (2%)

*Note:* MACS = Manual Ability Classification System; SD = standard deviation; ChARM = Children's Arm Rehabilitation Measure.

a Twelve parents reported children as lower limb impairment with no upper limb involvement; these children were included within the analyses.

Initial summary statistics for the ChARMs returned by the 148 parents are shown in Table 2 and indicated a degree of misfit to the Rasch model (chi‐square statistic = 128.9, *df* = 50, *p* < .001). Initial fit statistics for items suggested that only item 2, an “easy” item involving an activity of “pressing a button or switch,” displayed a significant misfit to the model.

**Table 2:** Summary statistics during the development of the final ChARM

Analysis	Item location		Person location		Item fit residual		Person fit residual		Chi‐square interaction			PSI		α	Unidimensionality *t* tests (CI)			
	Mean	SD	Mean	SD	Mean	SD	Mean	SD	Value	*df*	*p*	With extremes	NO extremes		Number of significant tests	Out of	%	Lower bound 95% CI
Initial analysis of draft 2	0.00	1.55	1.00	2.35	−0.41	0.95	−0.25	1.06	129	50	<.001	0.96	0.96	0.95	29	146	20	0.163
Final analysis draft 2	0.00	1.40	−0.65	2.99	−0.18	0.98	−0.20	0.78	46	38	.18	0.95	0.95	0.95	11	132	8	0.046

*Note.* ChARM = Children's Arm Rehabilitation Measure; CI = confidence interval; PSI = person separation index; SD = standard deviation. Initial analysis of draft 2: Initial summary statistics of Rasch analysis performed on one hundred forty‐eight 25‐item ChARM questionnaires returned for children described in Table 1.

Final analysis of draft 2: final summary statistics of Rasch analysis on 19‐item questionnaire after addressing psychometric issues.

### 3.2 Threshold ordering

**Figure 1:**
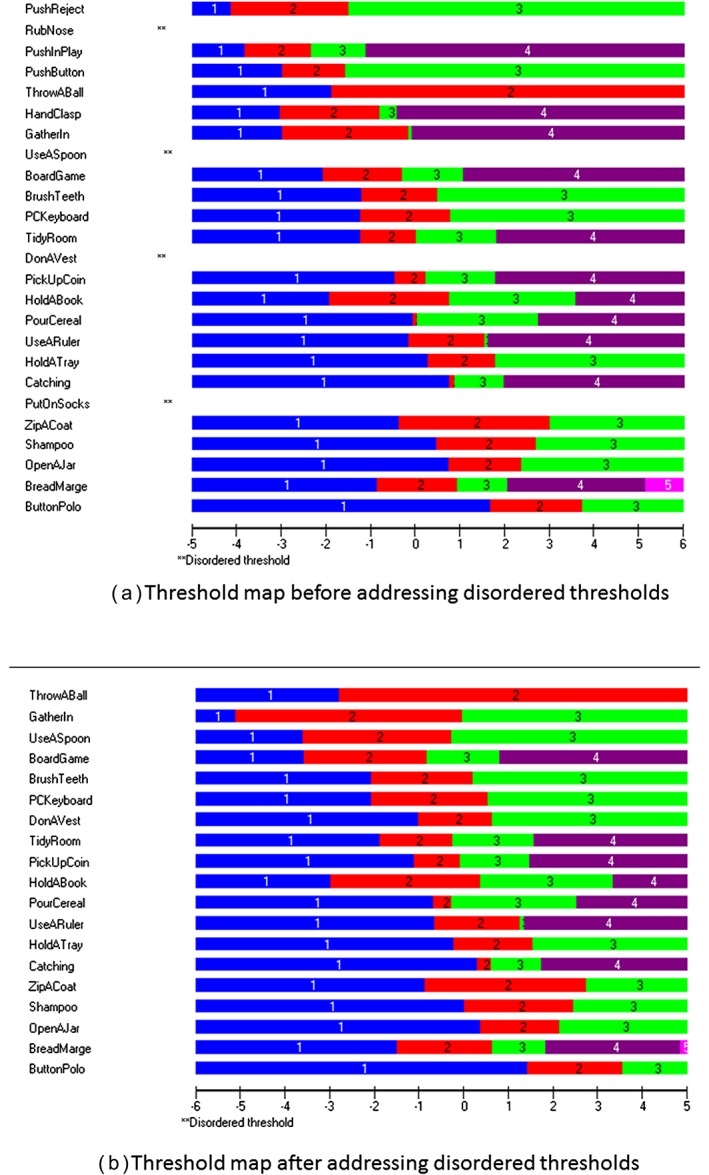
Threshold maps for the development of the Children's Arm Rehabilitation Measure during the final Rasch analysis

Five items initially displayed disordered response thresholds. To resolve this, we combined responses that illustrated disordered thresholds, using appropriate wording from each response to produce an ordered categorical response between the remaining unchanged categories. Figure 1 illustrates the threshold maps before and after addressing the disordered thresholds.

### 3.3 Local dependency and unidimensionality

A number of items displayed local dependence through a correlation of item residuals. All observed dependencies made conceptual sense, but the content of the dependent items did not lend themselves to the items being combined into a single item with a broader response range. We therefore resolved this issue in an iterative process by deleting the item, which displayed dependency with more items than any other, and then the dependent items with the least favourable fit statistics. After the removal of six items, including the misfitting item 2, the ChARM displayed acceptable evidence of unidimensionality (only 8% of *t* tests were significant, with the lower bound of the 95% confidence interval at 4.6%).

### 3.4 Item response bias

None of the items displayed any item response bias at a Bonferroni‐adjusted significance level, which was investigated for all items on the basis of age group (5–8 years old, 9–12 years old, or 13–16 years old), gender (male or female), distribution of arm impairment (unilateral or bilateral), learning difficulties, and visual impairment (present or not present).

### 3.5 Final summary item fit statistics

Final summary statistics are shown in Table 2, and final item fit statistics are shown in Table 3 for the final 19 items.

**Table 3:** Final ChARM item fit statistics

Item	Location	SE	Fit Residual	Χ^2^	*p*	Number of ordered response categories
12. Can your child throw a tennis ball (or a similar‐sized ball) to a catcher?	−2.784	0.33	−0.669	1.738	.42	2
1. Can your child gather in clothes, towels, blankets, or a soft toy with their arms and hands to clasp to their chest, either to hold for comfort or to carry?	−2.574	0.207	0.568	0.352	.84	3
8. Can your child feed themselves using a spoon?	−1.936	0.201	−1.44	7.605	.02	4
4. Can your child move pieces around a game board, for example, Snakes and Ladders, Draughts, Trivial Pursuit, Monopoly, Solitaire, or other board games?	−1.19	0.156	−0.34	0.267	.87	4
6. Can your child clean their own teeth, using any kind of toothbrush, if the toothpaste is put on the brush for them?	−0.926	0.183	−0.04	0.887	.64	3
5. Can your child use a computer keyboard?	−0.758	0.184	1.44	0.19	.91	3
14. Can your child put on a vest (or short‐sleeved T‐shirt—do not worry about buttons) if it is laid out properly for them?	−0.178	0.169	−1.603	1.605	.45	3
16. Can your child tidy their bedroom?	−0.178	0.175	−0.98	0.135	.93	6
2. Can your child pick up a coin from a table with one hand and put it into a purse or wallet held in the other arm or hand?	0.099	0.139	1.149	1.668	.43	4
18. Can your child use both hands when writing or drawing, for example, one hand to write or draw and the other to hold the book open or the paper still?	0.26	0.164	1.484	2.097	.4	4
9. Can your child pour breakfast cereal into a bowl from a box of cereal that is already open (e.g., Cheerios, Frosties and Cornflakes)?	0.531	0.142	−0.557	0.234	.89	4
15. Can your child use a ruler for drawing and for underlining words?	0.661	0.132	0.12	2.403	.3	4
17. Can your child pick up and hold a plate or tray of food?	0.667	0.166	−0.991	1.4	.5	3
13. Can your child catch something thrown from 3 steps away?	0.894	0.129	0.72	9.209	.01	4
11. Can your child zip up a coat by themselves?	0.932	0.187	−0.89	2.149	.34	3
19. Can your child apply hair products to their hair independently (e.g., shampoo or hair gel)?	1.244	0.174	−0.982	2.457	.29	3
7. Can your child open a previously opened jar of spread, for example, chocolate spread, peanut butter, or jam?	1.267	0.169	−1.136	6.757	.03	3
10. Can your child spread butter (or margarine) on a slice of bread?	1.466	0.135	0.876	2.364	.31	5
3. Can your child button a polo shirt (one that only has a few buttons)?	2.502	0.194	−0.224	2.182	.34	3

*Note.* SE = standard error.

Once the final psychometrically acceptable item set had been established, deleted items were reintroduced, one at a time, to the final item set to check that the initial misfit anomaly was still present. This was the case for all deleted items, so none were included in the final item set.

**Figure 2:**
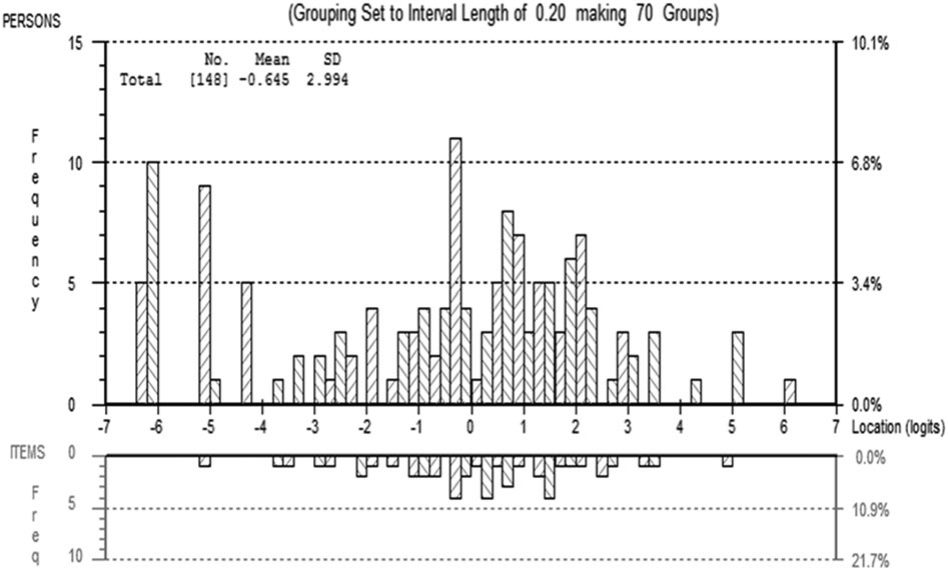
Person–item distribution for the final version of the Children's Arm Rehabilitation Measure

A person–item distribution map is shown in Figure 2.

Sixteen children (12%) fell outside of the measurement range of the scale (15 children with the greatest arm activity limitation and one child with the least arm activity limitation), therefore the calibration (and associated analysis results) is based on 132 children's data. This proportion of children does not represent a floor or ceiling effect (Holmes & Shea, 1997). Of the extreme scores, one was from a questionnaire returned with no demographics or clinical data, 13 were MACS Level V, and one was MACS Level IV. Of the 14 with clinical data, all had learning disability and all but one had bilateral arm impairment. Five children of MACS Level V were represented on the scale, and these children also had learning disability. All but one of the 45 children with MACS Level IV were represented on the scale.

This sample size also provides at least 95% confidence of item calibration to within 0.5 logit, although given the good targeting parameters of the scale, it is likely that a 99% confidence of item calibration to within 0.5 logit has been achieved (Linacre, 1994).

### 3.6 Reliability

The PSI illustrates an internal consistency of 0.946 (extremes included and 0.951 without extremes, see Table 2). The Cronbach's α value of the final item set is 0.95, indicating good targeting of the item distribution.

### 3.7 Construct validity

The results of an analysis of variance performed on mean logit scores for all children within each MACS level showed a significant difference (F [4,141] = 121.1, *p* < .001) between scores (MACS Level I, 3.766 [SD 1.43]; Level II, 2.099 [SD 1.2]; MACS Level III, 0.429 [SD 1.01]; MACS Level IV, −2.131 [SD 1.94]; and MACS V, −5.185 [SD 0.59]), suggesting good external construct validity.

## 4 DISCUSSION

This study successfully developed a psychometrically robust measure of upper limb activity limitation specifically validated for children with cerebral palsy aged 5–16 years. The ChARM is unidimensional, has excellent reliability, and displays no response bias for gender, topography, age or learning disability, and no floor or ceiling effects. The sample size permitted a strong calibration of items (Linacre, 1994).

Post‐development psychometric testing to establish the measurement properties of a new measure is essential (Hobart et al., 2007; Tennant, 2007), but nothing in this subsequent validation can rectify badly selected and inappropriate items (Streiner & Norman, 2003, p. 15). Defining and selecting items that adequately represent the characteristic to be measured are of critical importance (Wilson, 2005, p. 64). Diligent design of outcome measures may help to prevent limitations described above (Hobart et al., 2007), starting with careful consideration of the actual trait being measured (Hobart et al., 2007). Our strategy for developing items ensured that items would be valid and appropriate for a high proportion of children with cerebral palsy across the targeted age and manual ability range and crucially that would represent the single characteristic that was to be evaluated: changes to upper limb activity limitation, as defined by the World Health Organisation (World Health Organisation, 2002; World Health Organisation, 2016). We will evaluate responsiveness in a subsequent study.

We decided against a standard (identical) response format for each item and elected to include all potentially appropriate response options knowing that they would be evaluated empirically, using Rasch analysis to identify disordering of thresholds and demonstrate which response options were working as intended. Although this approach means that the ChARM will be more time‐consuming to complete for respondents (because each item has different responses to read and consider), it offers several advantages: the optimal number of response options has been generated for each item (Bond & Fox, 2015, p. 160); it facilitates easy identification of stages of achievement, which a child has reached (Bobath, 1990); it avoids the potential uncertainty for the respondent of which response option to endorse that occurs with homogenous item response options; and it overcomes the halo effect (when respondents endorse the same response category for each item; Streiner & Norman, 2003, p. 39). No parent reported taking longer than 4 min to complete the ChARM.

### 4.1 Limitations

Despite the promising results, some study limitations are present. Developments of the items were from goals of activity rehabilitation taken from 53 children. This was a smaller number than anticipated, given that the therapy teams involved covered well‐populated areas potentially including up to 2,000 children with cerebral palsy, none of whom was excluded outside of the age range 5–16 years old. Possible reasons for this poor initial recruitment include the requirement of all participants in the early stages to give full, written, informed consent to take part in the study despite the low impact of the study on children's care. This has now been recognised by the National Health Service research ethics service, and proportionate review is now available for studies of this nature. However, the ethics committee removed the need for written consent because the data were all anonymised, and a return of the questionnaire to researchers by parents was considered by the ethics committee to imply that informed consent was given. Additionally, an unknown number of parents were excluded on the basis of the judgement of participating therapists. However, although this number of children is smaller than anticipated, 78 unique goals delivered a wide range of appropriate activity‐related categories, and the final item set has a broad range of activities and includes items, which are potentially achievable by some of the most disabled children with cerebral palsy, thus overcoming the floor effect. Although we are unlikely to have received the full range and breadth of goals that we had hoped, recent studies investigating the efficacy of new approaches to reducing activity limitation have independently identified the same or similar goals (Sakzewski et al., 2011; Wallen, O'flaherty, & Waugh, 2007), suggesting that our efforts to identify the most common activity limitations in children with cerebral palsy met with some success.

Although therapy teams were asked to send the ChARM to parents of children exhibiting upper limb activity limitation, we received ChARMs for 12 children described as MACS Level I. Because children with cerebral palsy often exhibit mild impairment in all four limbs even when categorised with unilateral or lower limb impairment, we included all children in the analyses. This decision seems to have been justified because only one child with MACS Level I fell outside the upper extreme of the measurement range, only five (excluding the extreme child) fell in the top 10 performers, and the worst performing MACS I child was 31st in descending order of the 148 children. This suggests that the ChARM could be sensitive enough for the evaluation of activity limitation of children with even a mild movement disability.

The scale reliability (internal consistency) presented by the PSI and Cronbach's α is very high, each at a value of 0.95. This value meets the standard required for use at the individual level.

## 5 IMPLICATIONS FOR PHYSIOTHERAPY PRACTICE

The development of the new measure presented here used a novel method and was designed to address limitations in other measures. It is based on the most common clinically relevant goals for the population on which it is validated. The ChARM is validated for completion by parents or carers without the guidance of health care professionals and can be completed at home (through postal services) or in health service waiting rooms. It is designed to evaluate change in independent arm activity limitation following any intervention for that purpose in children with cerebral palsy.

The use of appropriate, valid measurement scales for accurately identifying outcomes of intervention programmes is essential in research and clinical practice. This paper provides a template for the development of other psychometrically sound measures that have both clinical and scientific validation and significance.

The ChARM can be freely obtained from the Academic Department of Rehabilitation Medicine at the University of Leeds, West Yorkshire, United Kingdom. This requires registration with the Psychometric Laboratory staff through the website http://medhealth.leeds.ac.uk/info/732/psychometric_laboratory/. Registration and use of the ChARM are free to clinicians and nonprofit organisations.
